# Analysis of Short-Term Smoking Effects in PBMC of Healthy Subjects—Preliminary Study

**DOI:** 10.3390/ijerph15051021

**Published:** 2018-05-18

**Authors:** Joanna Wieczfinska, Tomasz Kowalczyk, Przemyslaw Sitarek, Ewa Skała, Rafal Pawliczak

**Affiliations:** 1 Department of Immunopathology, Medical University of Lodz, Lodz 90-752, Poland; j.wiecz@csk.umed.lodz.pl; 2 Department of Genetics and Plant Molecular Biology and Biotechnology, The University of Lodz, Lodz 90-237, Poland; tomasz.kowalczyk@biol.uni.lodz.pl; 3 Department of Biology and Pharmaceutical Botany, Medical University of Lodz, Lodz 90-151, Poland; przemyslaw.sitarek@umed.lodz.pl (P.S.); ewa.skala@umed.lodz.pl (E.S.)

**Keywords:** cigarette smoking, PBMC, COPD, airway remodeling, apocynin, oxidative stress

## Abstract

Early structural changes exist in the small airways before the establishment of Chronic Obstructive Pulmonary Disease (COPD). These changes are believed to be induced by oxidation. The aim of this study was to analyze the influence of short-term smoking on the expression of the genes contributing to airway remodeling and their relationship with the oxidative status of human blood cells. Blood mononuclear cells were isolated from 16 healthy volunteers and treated with cigarette smoke ingredients (CSI): nicotine, 1-Nitrosodimethylamine, N-Nitrosopyrrolidyne, vinyl chloride, acetone, and acrolein. The expression of TGF-β1, TIMP-1, SOD1, and arginase I was determined by qPCR. Additionally, thiol groups and TBARs were assessed. CSI induced TGF and TIMP-1 expression in peripheral blood mononuclear cells (PBMC), and apocynin alleviated this effect. The changes were more noticeable in the smoking group (*p* < 0.05). TBARs concentrations were higher in smokers, and in this group, apocynin acted more effectively. SOD1 correlated with arginase expression in smokers (*p* < 0.05). MMP-9 showed a significant correlation with SOD1 in both groups, but only on the protein level. Blood cells appear to mirror the general changes caused by cigarette smoke ingredients, which seem to be connected with the oxidative status of the cell. Our findings indicate that a short period of smoking influences the gene expression and oxidative balance of blood cells, which might result in the development of serious disorders such as COPD.

## 1. Introduction

Cigarette use is one of the most important causes of early and preventable death and a significant public health concern worldwide [1]. The smoke contains more than 4000 harmful chemical compounds, of which 200 are very toxic, with 1017 free radicals/oxidants present in each puff [2,3]. These compounds are responsible for the recruitment of inflammatory agents and cells and the formation of oxidants, thus inducing oxidative stress and the oxidation of proteins and thiol groups [4].

Smoking is a widespread behavior among adolescents and a major subject of discussion all around the world. It has been reported that up to 15% of all smokers and up to 26% of heavy smokers develop Chronic Obstructive Pulmonary Disease (COPD), typically characterized by persistent airflow obstruction and chronic inflammation of the airways [5,6,7,8]. However, the changes leading to serious disorders (increase of reactive oxygen species, increase of alveolar cellularity, with a higher proportion of neutrophils and a smaller proportion of lymphocytes) may start after only a short period of smoking [2,9,10].

Oxidative stress is defined as a disturbance in the balance between the production of free radicals such as reactive oxygen species (ROS) and antioxidant defenses [11,12]. It has a negative effect on cellular proteins (modify redox-sensitive amino acids in a variety of proteins, including phosphatases, ion channels, and transcription factors, oxidation of proteins may lead to the formation of insoluble protein aggregates, it can cause oxidation of the protein backbone resulting in the protein fragmentation, and formation of protein–protein cross-linkages), despite the existence of protective systems and reductive pathways. The accumulation of oxidized protein products impairs the function of cells and may even lead to cell death. During oxidative stress, oxidation of cellular thiol (-SH) groups is observed through the direct effect of reactive oxygen species (ROS). Oxidative damage causes a rapid loss of biological activity of the protein, leads to disorders in the proper functioning of many transporters and enzymes and disrupts the calcium homeostasis [13,14].

One of the main sources of reactive oxygen species (ROS) in cells is NADPH oxidase [15]. Cigarette smoke is known to enhance the expression of the gp91phox gene, which is one of the components of the NADPH oxidase complex. Apocynin can effectively inhibit NADPH oxidase activity and thus reduce the negative influence of cigarette smoke on subsequent ROS generation [16].

Airway remodeling involves various gradual structural changes being made to a range of tissues, which are necessary for correct development during embryogenesis and organogenesis [17]. However, in the pathophysiology of the respiratory tract, airway remodeling concerns highly composed structural transformations, which affects the airways and leads to significant functional impairment [18,19]. Although airway remodeling is known to be associated with the effects of cigarette smoke—induced inflammation in the airway wall, its pathogenesis remains poorly understood.

Therefore, the aim of the current preliminary study was to determine the relationship between cigarette smoking, the genes related to airway remodeling and oxidative stress. It also evaluates the effect of the components of cigarette smoke and apocynin, an NADPH inhibitor, on gene expression in smoking and nonsmoking groups of subjects.

## 2. Materials and Methods

### 2.1. Patients

The study was conducted on 16 systematically healthy subjects (eight men and eight women, mean age 24 years) who were divided into two groups: smoking and nonsmoking (eight subjects each) (Table 1). The project was approved by Bioethical Committee for Research Studies of the Medical University of Lodz (ethics approval number: RNN/62/16/KE) and written informed consent has been obtained from the enrolled subjects.

The group of smokers included current and moderate smokers reporting consumption of between 10 and 20 cigarettes per day for at least three years. The subjects were excluded from the study if their medical conditions required drug therapy or administration of nonsteroidal or anti-inflammatory drugs. The subjects were asked not to use coffee, green tea, or vitamins a week before the study and not to smoke for six hours before sample collection.

### 2.2. Blood Collection, PBMCs, and Serum Isolation

Venous blood (28 mL) was collected and peripheral blood mononuclear cells (PBMC) were isolated by centrifugation using a Histopaque^®^ 1077 solution (Sigma Aldrich, Saint Louis, MO, USA) according to the manufacturer’s protocol and washed three times in PBS. Part of the blood (one sample from each volunteer) was allowed to clot for 30 min at room temperature and centrifuged at 2000× *g* for 15 min at 4 °C to obtain serum.

Since cigarette smoke consists of compounds known to be mutagenic, carcinogenic, antigenic, and cytotoxic, the effect on cell viability of chosen cigarette smoke ingredients (CSI) concentrations used in this study was tested using trypan blue (Table 2).

### 2.3. Experimental Design

The cells isolated from the blood were plated at 2 × 10^6^ cells per well, cultured for 24 h in Dulbeccos’s modified eagle medium (DMEM) containing 10% fetal bovine serum (FBS), penicillin (100 units/mL) and streptomycin (100 ug/mL). Subsequently, the cells were treated with apocynin (2.5 mg/mL) and/or the following cigarette smoke ingredients (CSI) for 24 h at 37 °C: nicotine—2.5 mg, 1-Nitrosodimethylamine—53.75 ng, N-Nitrosopyrrolidyne—36.25 ng, vinyl chloride—17.4 ng, acetone—21.3 ng, and acrolein—9.5 ng. The concentrations were calculated to be representative of the amounts present in one cigarette.

Each experiment was performed 4 times, the groups were marked as Nsm—nonsmoking group and Sm—smoking group.

### 2.4. RNA Extraction and cDNA Synthesis

Total RNA was isolated from the stimulated cells using RNeasy Cell Mini Kits with QIAshredder (Qiagen, Hilden, Germany). RNA was DNase treated, purified, eluted in 30 μL of RNase-free water and stored at −80 °C for further analysis. Total RNA (1 μg) was reverse-transcribed using High Capacity cDNA kit (Applied Biosystems, Foster City, CA, USA). All procedures were carried out as indicated by the manufacturer. 

### 2.5. Analysis of Gene Expression

Real-Time PCR was conducted in order to evaluate the changes in expression of TIMP-1, TGF-β1, MMP-9, SOD1 and arginase-I genes. cDNA was subjected to qPCR using the assays designed for the selected genes (Hs00171558_m1 for TIMP-1, Hs00968979_m1 for arginase I, and Hs00998133_m1 for TGF-β1) and β-actin (Hs99999903_m1), as a qPCR reference (Life Technologies, Carlsbad, CA, USA). Each sample was measured in triplicate using TaqMan analyzer 7900 (Life Technologies, Carlsbad, CA, USA). Using the 2-ΔΔCt method, the results are presented as gene expression normalized to an endogenous reference gene (β-actin) and relative to a control. The fold change of mRNA expression in each patient was calculated by comparing RQ (2-ΔΔCt).

### 2.6. Estimation of SOD Level

Activity of extracellular SOD in cell lysates was measured with a SOD assay kit (Cayman Chemicals, Ann Arbor, MI, USA) according to the manufacturer’s protocol. For analysis, 10 µL serum samples were used; the reaction was initiated by adding 20 μL of xanthine oxidase. The plate was mixed for a few seconds, covered and incubated on a shaker for 30 min at room temperature. Absorbance was read at 450 nm in a microplate reader. SOD activity was expressed as U/mL. One unit of SOD activity was defined as the amount of enzyme needed to exhibit 50% dismutation of the superoxide radical.

### 2.7. Estimation of Thiol Groups

Total thiol content was determined using the 5,50-dithiobis (2-nitrobenzoic acid)-DTNB-method (Sigma, St. Louis, MO, USA) using conditions previously described [20]. Briefly, 30 µL of a sample was mixed with 1 mL of PBS and 1 mM of EDTA (pH 7.5). Addition of 30 µL of 10 mM DTNB stock solution in PBS started the reaction and incubated for 30 min at room temperature. Control samples, without DTNB, were run simultaneously. The absorbance was read at 412 nm, and the amounts of 5-thio-2-nitrobenzoic acid (TNB) formed (equivalent to the amount of sulfhydryl groups) were measured.

### 2.8. Estimation of TBARs

The level of lipid peroxidation was measured using the TBARS assay kit (Cayman Chemical, Ann Arbor, MI, USA) in serum and in PBMCs lysates according to the manufacturer’s instructions. The principle of this assay assumes that malondialdehyde (MDA) and thiobarbituric acid (TBA) form an MDA-TBA adduct in conditions of high temperature and acid conditions. This adduct might be measured colorimetrically (530 nm). Total TBARS, as a proxy for lipid peroxidation (malondialdehyde levels), are expressed as mM.

### 2.9. Statistical Analysis

All results were expressed as mean ± standard deviation (SD). Groups were compared using the Student’s *t*-test and Mann-Whitney rank sum test, depending on the distribution of the obtained data. Correlation studies were performed using the Pearson Correlation test. A *p*-value lower than 0.05 was considered statistically significant for all tests.

## 3. Results

### 3.1. PBMCs of Smoking Subjects Respond with Higher mRNA Expression

**Figure 1:**
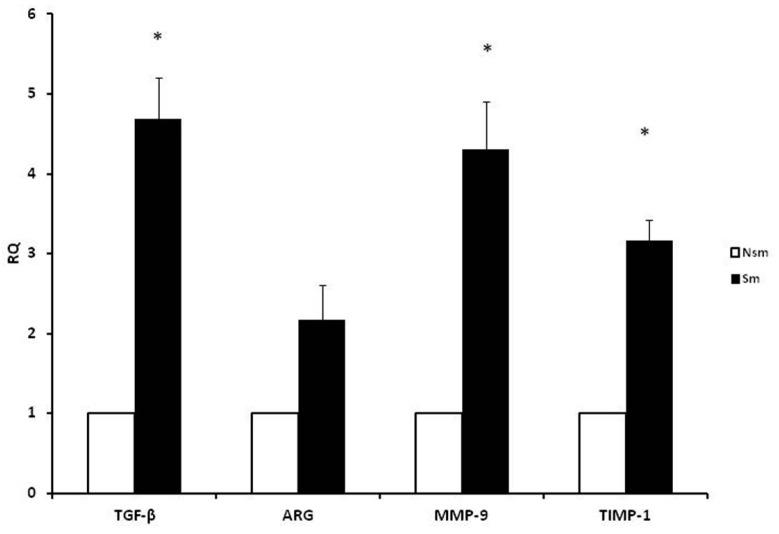
Results of qPCR. Expressions of TGF-β, arginase, MMP-9, and TIMP-1 were increased in smokers in comparison to never-smokers. Data obtained from peripheral blood mononuclear cells (PBMC), presented as RQ ± SD. Nsm—nonsmoking group, Sm—smoking group. *p* = 0.032 for TGF-β, *p* = 0.038 for MMP-9, *p* = 0.045 for TIMP-1.

To determine the influence of apocynin and cigarette smoke ingredients on the mRNA expression of the genes contributing airway remodeling, a real-time PCR reaction was performed. First, we confirmed the differences in the mRNA expression of chosen genes was compared between the smoking and the never-smoked group. The results, presented as RQ, clearly show that the smoking subjects presented lower expression of the analyzed genes connected with airway remodeling (Figure 1).

### 3.2. Results of qPCR in Smoking and Nonsmoking Groups

We then examined the influence of the selected CSI and apocynin, an NADPH inhibitor, both alone and in combination, on the expression of genes involved in the remodeling process.

**Figure 2:**
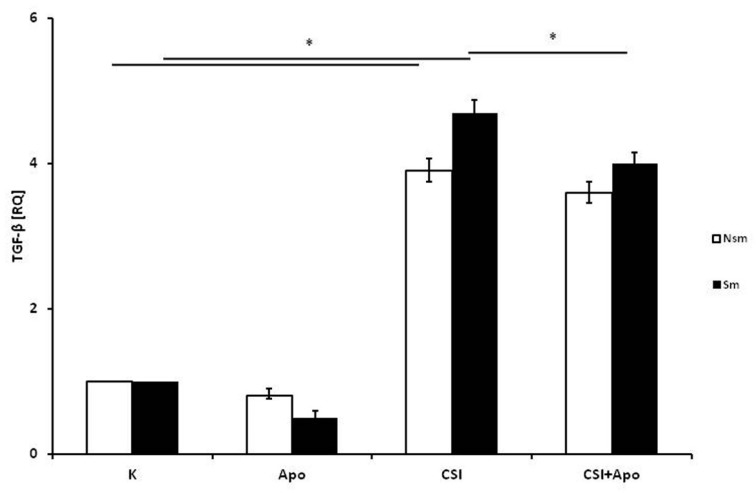
Expression of TGF-β mRNA in response to cigarette smoke ingredients (CSI) and apocynin. CSI induced TGF-β expression in the PBMC of both groups (*p* < 0.05). In smokers, this effect was reduced by apocynin. White bars represent the nonsmoking group and the black bars the smoking group. Data is presented as relative expression (RQ) ± SD, * *p* < 0.05. CSI included nicotine—2.5 mg, 1-Nitrosodimethylamine—53.75 ng, N-Nitrosopyrrolidyne—36.25 ng, vinyl chloride—17.4 ng, acetone—21.3 ng, and acrolein—9.5 ng.

Real-time PCR found that a statistically significant increase in TGF-β mRNA expression in both groups (*p* < 0.05), with the PBMC from smoking subjects demonstrating stronger induction of TGF-β mRNA than those from the nonsmoking group (Figure 2). Moreover, this difference was also significant between groups (*p* < 0.05). Apocynin application resulted in an insignificant decrease of TGF-β mRNA expression. Apocynin application slightly decreased the effect of CSI, but this was significant only in the smoking group (*p* < 0.05).

**Figure 3:**
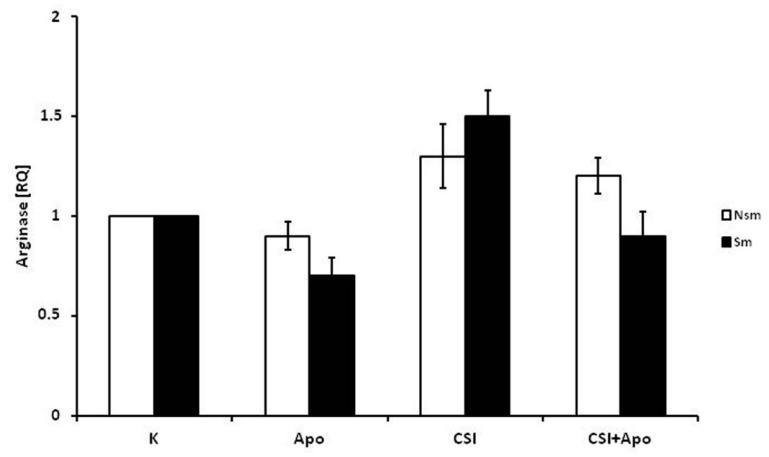
Relative expression of arginase after CSI and apocynin stimulation. No statistically significant response was observed to CSI and apocynin treatment in PBMC from either the smoking or the non-smoking group. The white bars represent the non-smoking group, and the black bars the smoking group. Data is presented as relative expression (RQ) ± SD. CSI included nicotine—2.5 mg, 1-Nitrosodimethylamine—53.75 ng, N-Nitrosopyrrolidyne—36.25 ng, vinyl chloride—17.4 ng, acetone—21.3 ng, and acrolein—9.5 ng. Data presented are statistically insignificant.

Apocynin and CSI were observed to have a similar effect on the expression of arginase I mRNA (Figure 3), with apocynin insignificantly decreasing, and CSI insignificantly increasing, the expression of arginase I in both groups (*p* > 0.05). However, apocynin showed a tendency to alleviate the effect of CSI by returning the level of expression to baseline (*p* > 0.05). Interestingly, in the nonsmoking group, apocynin was observed to have no effect.

**Figure 4:**
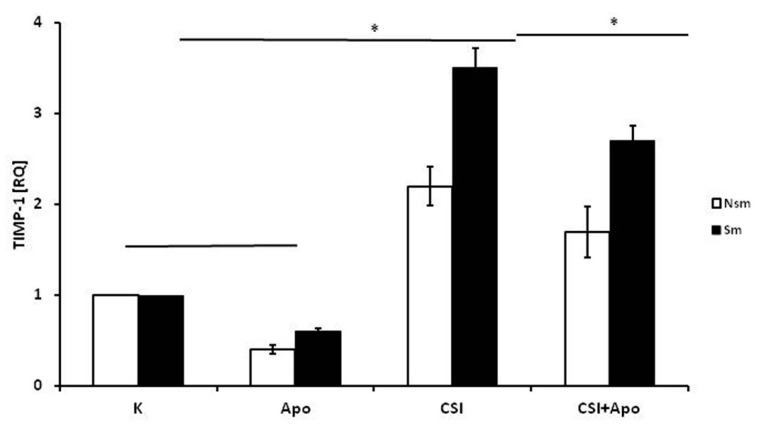
The effect of CSI and apocynin on relative expression of TIMP-1 by PBMC of smoking and non-smoking subjects. Apocynin decreased TIMP-1 expression in the in PBMC of the non-smoking group and diminished CSI-induced expression significantly in the smokers. White bars represent the non-smoking group, and the black bars the smoking group. Data is presented as relative expression (RQ) ± SD, * *p* < 0.05. CSI included nicotine—2.5 mg, 1-Nitrosodimethylamine—53.75 ng, N-Nitrosopyrrolidyne—36.25 ng, vinyl chloride—17.4 ng, acetone—21.3 ng, and acrolein—9.5 ng.

TIMP-1 mRNA expression differed between smoking and nonsmoking subjects (Figure 4). Both groups demonstrated changes in expression after CSI incubation (RQ = 2.2 for smokers and 3.5 for non-smokers), but this change was significant only in smokers (*p* < 0.05). Also, apocynin significantly inhibited the effect of CSI in smokers (*p* < 0.05). Surprisingly, apocynin alone decreased TIMP-1 mRNA expression (*p* < 0.05) in the PBMC of nonsmoking subjects.

**Figure 5:**
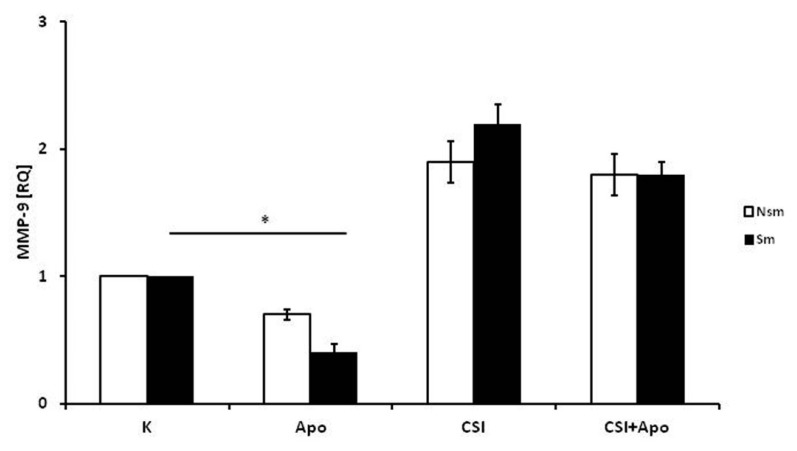
MMP-9 expression in PBMC of smokers and nonsmokers. Apocynin caused a significant decrease in MMP-9 expression in smoking subjects, but had no significant influence on CSI-induced expression of MMP-9. The white bars represent the nonsmoking group, and the black bars the smoking group. Data is presented as relative expression (RQ) ± SD, * *p* < 0.05. CSI included nicotine—2.5 mg, 1-Nitrosodimethylamine—53.75 ng, N-Nitrosopyrrolidyne—36.25 ng, vinyl chloride—17.4 ng, acetone—21.3 ng, and acrolein—9.5 ng.

In contrast, a significant decrease was observed in MMP-9 mRNA expression after incubation with apocynin in the smoking group (RQ = 0.4, *p* < 0.05) (Figure 5). In addition, apocynin did not show clear tendency to decrease CSI-induced expression of MMP-9 in either group (*p* > 0.05). In addition, no significant increase of MMP-9 mRNA expression was observed after CSI induction (*p* > 0.05).

### 3.3. Oxidative and Antioxidative Properties of PBMC in Smokers and Never Smokers

**Figure 6:**
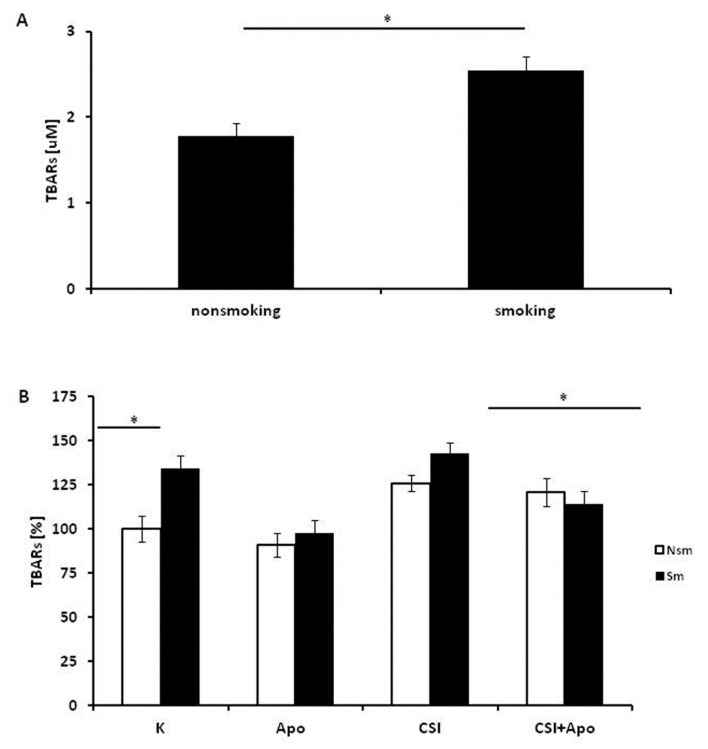
TBAR levels in serum and PBMC lysates of smoking and non-smoking subjects. TBAR levels, measured as MDA, were significantly higher in smokers than in nonsmokers, both in serum (**A**) and in cell lysates (**B**). Apocynin had a significant soothing effect in the smoking group (*p* < 0.05) but not in the nonsmoking group (*p* > 0.05) (**B**). In part B, the white bars represent the non-smoking group, and the black bars the smoking group. Data is shown as mean ± SD, * *p* < 0.05. CSI included nicotine—2.5 mg, 1-Nitrosodimethylamine—53.75 ng, N-Nitrosopyrrolidyne—36.25 ng, vinyl chloride—17.4 ng, acetone—21.3 ng, and acrolein—9.5 ng.

**Figure 7:**
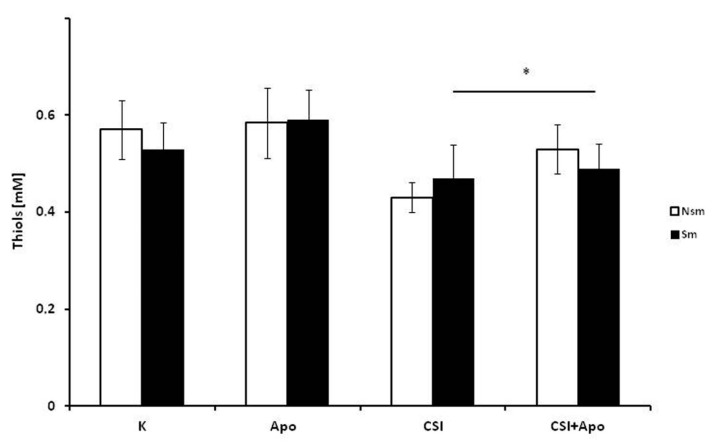
The effect of CSI and apocynin on PBMCs incubated with CSI and apocynin in the smoking and non-smoking groups. Apocynin increased CSI-reduced thiol content; however, this change was significant only in the non-smoking group. The white bars represent the non-smoking group, and the black bars the smoking group. Data is presented as mean ± SD, * *p* < 0.05. CSI included nicotine—2.5 mg, 1-Nitrosodimethylamine—53.75 ng, N-Nitrosopyrrolidyne—36.25 ng, vinyl chloride—17.4 ng, acetone—21.3 ng, and acrolein—9.5 ng.

**Figure 8:**
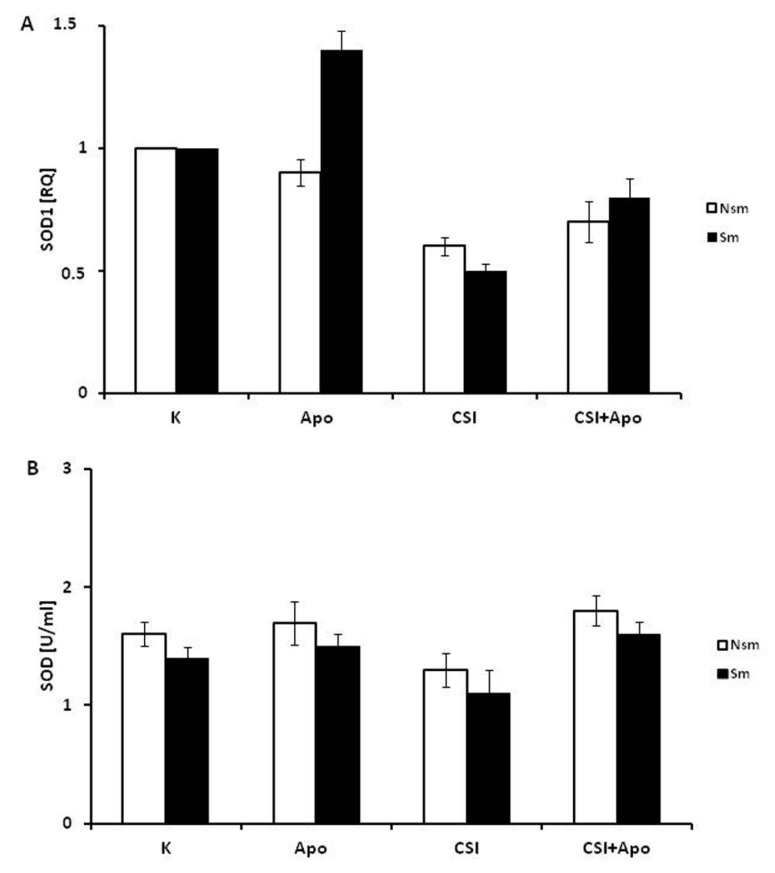
Superoxide dismutase 1 (CuZnSOD) expression on mRNA (**A**) and protein (**B**) levels in PBMCs of smoking and non-smoking patients. CSI reduced SOD1 expression in both groups, and the effect was softened by apocynin. The changes were clearer at mRNA level (A); however, they were not statistically significant. White bars represent the non-smoking group, and the black bars the smoking group. Data is presented as mean ± SD. CSI included nicotine—2.5 mg, 1-Nitrosodimethylamine—53.75 ng, N-Nitrosopyrrolidyne—36.25 ng, vinyl chloride—17.4 ng, acetone—21.3 ng, and acrolein—9.5 ng. Data presented are statistically insignificant.

The next stage evaluated the influence of CSI and apocynin on TBAR, thiol and SOD1 concentration and SOD1 expression in both groups. Differences in thiol, TBARs and SOD1 concentrations were observed at baseline between smokers and non-smokers (Figure 6, Figure 7 and Figure 8).

The smokers showed higher TBAR levels in serum than non-smokers (*p* < 0.05) (Figure 6A). Apocynin slightly decreased the concentration of TBARs in cell lysates when applied alone (*p* > 0.05) but reduces the effect of CSI when applied in combination. Interestingly, while this trend occurred in both groups, the change was only significant in smokers (*p* < 0.05) (Figure 6B). 

While apocynin increased PBMC thiol concentrations following their reduction by CSI in both groups, this change was only significant in non-smoking subjects (*p* < 0.05) (Figure 7). This effect of apocynin was not obtained in respect to SOD1 expression neither on mRNA nor protein level. CSI decreased SOD1 expression, and the effect was leveled off by apocynin (Figure 8A). In addition, the application of apocynin alone caused an insignificant increase of SOD1 expression in the smoking group. However, these findings were not reflected in the SOD1 protein levels (Figure 8B). 

### 3.4. Superoxide Dismutase 1 Correlates with Arginase and MMP-9 mRNA Expression

To identify any possible connection between early cigarette smoking and the occurrence of COPD and remodeling, the next stage of the study examined the relationship between the expression of selected genes involved in the airway remodeling process and certain parameters of oxidative stress and antioxidative capacity of the tested cells.

As RQ values from nonstimulated cells cannot be compared in this case, deltaCt was used to compare the identified expression with oxidative stress parameters.

**Figure 9:**
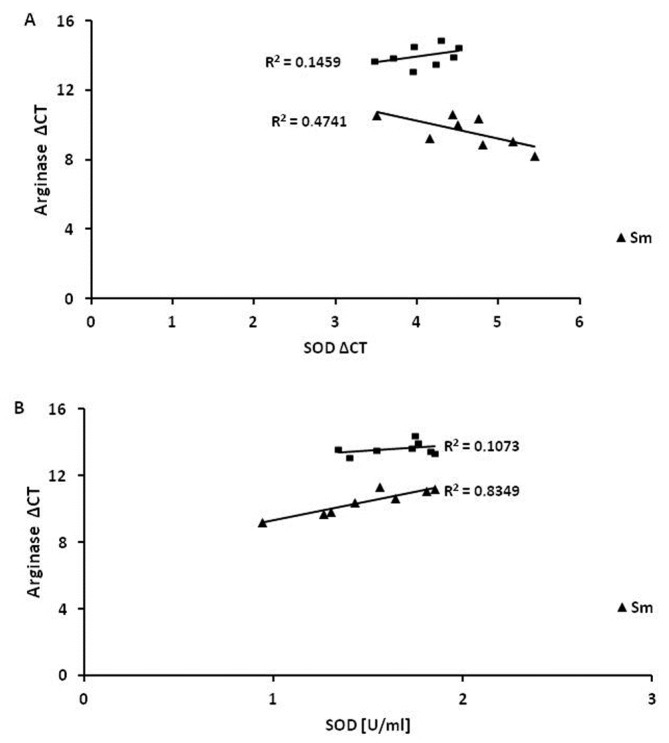
Pearson correlation coefficient analysis. (**A**) presents the correlation between arginase mRNA expression (presented as deltaCT) and CuZnSOD mRNA expression (delta CT). In both tested groups, the correlation coefficient was significant (*p* < 0.05). However, it was stronger in smokers than nonsmokers (−0.6 vs. 0.3 respectively, *p* < 0.05). (**B**) shows the correlation between arginase mRNA expression (presented as deltaCT) and CuZnSOD mRNA expression. The Pearson coefficient was statistically significant for smokers (0.91) (*p* < 0.05), but not for non-smokers (0.3, *p* > 0.05).

The Pearson correlation coefficient indicated a fairly strong correlation between arginase expression and concentrations of SOD1 in the smoking group (Table 3). The correlation was positive, but interestingly, this correlation was opposite in smoking (0.91; *p* < 0.05) and nonsmoking subjects (0.3; *p* > 0.05). The expression data were deltaCt, as the relative quantity at the baseline was always 1. Therefore, in the smoking group, higher expression of arginase mRNA was connected with lower expression of SOD1 (Figure 9A). This relationship was found to be much less significant in the non-smoking group.

Surprisingly, a further analysis of the correlation between deltaCT of arginase and SOD1 mRNA found an opposite correlation in smokers and nonsmokers, although it was not as high as in case of SOD1 protein (−0.6 and 0.38 respectively, *p* < 0.05) (Figure 9B). 

**Figure 10:**
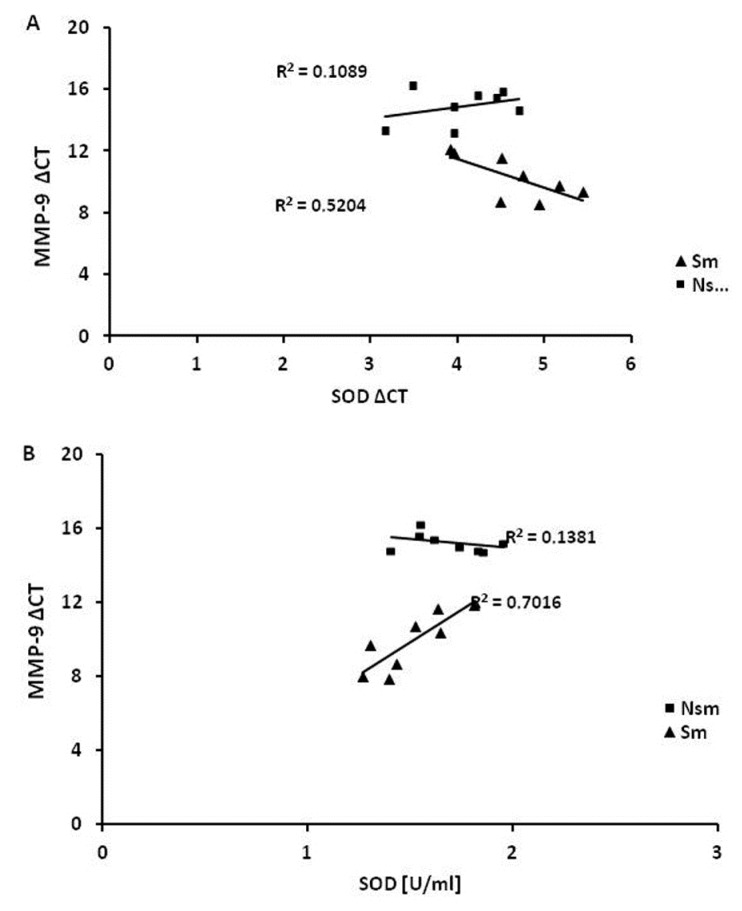
Pearson correlation coefficient analysis. Correlation between MMP-9 and SOD1 expression on the mRNA level (deltaCT) gave no significant results (smoking −0.72 and nonsmoking 0.33, *p* > 0.05) (**A**). (**B**) presents a correlation between MMP-9 deltaCT and CuZnSOD protein expression. The correlation coefficient was significant in both groups (*p* < 0.05) but was stronger in smokers than in nonsmokers (0.83 vs. −0.3 respectively, *p* < 0.05).

Pearson analysis also revealed a relationship between MMP-9 expression and SOD concentration in cell lysates. However, while occurring in both groups, it was opposite (smoking group 0.83, nonsmoking group −0.3, *p* < 0.05), (Figure 10A,B). The results from smoking volunteers show that the lower deltaCt, the lower SOD1 concentration, indicating that higher relative expression of MMP-9 is correlated with lower concentrations of SOD1 (*p* < 0.05). 

A weak correlation was found between MMP-9 and SOD1 mRNA levels (Figure 10A). However, while the correlation was weak in the PBMCs of the non-smoking group (0.33, *p* < 0.05), a strong, negative correlation was observed in those of the smokers (−0.72, *p* < 0.05).

## 4. Discussion

Tobacco smoking is the most common risk factor for chronic obstructive pulmonary disease, a disease that has been projected to become the third leading cause of death globally by 2030 [21]. Although COPD is highly prevalent in the global population of older adults, i.e., those aged 40 years and above, irreversible changes occur in the airways possibly many years before its development. The present study compares two groups of young adults to identify the potential changes occurring after just a few years of smoking.

The common belief is that the process of airway remodeling derives from long-term smoke-induced chronic inflammation in the airway walls [22,23,24]. Our current findings indicate that short-term periods of smoking induce different gene expression patterns from those of non-smoking subjects: the mRNA expression of all evaluated genes, i.e., TGF-β, arginase, MMP-9 and TIMP-1, was found to increase in response to exposure to cigarette smoke ingredients (CSI). These results may indicate that the changes leading to the development of COPD start as soon as after a few years of smoking, and are consistent with the literature; however, most previous studies on this area use older study groups [25,26].

Interestingly, our study shows that the PBMCs of smoking and nonsmoking subjects react differentially to stimulation with CSI: in each of the genes analyzed, the expression was higher in smokers than in nonsmokers, which might suggest that the cells of the smokers are more sensitive to cigarette smoke ingredients [27]. Smoking may cause substantial change in the distribution of lymphocyte subtypes and impairment of lymphocyte function [28]. Wang, R.D. et al. report that cigarette smoke can directly induce airway remodeling, specifically airway wall fibrosis, probably through reactive oxygen species-dependent transactivation of the epidermal growth factor receptor and subsequent nuclear factor-κB activation [22]. There is limited information on the in vitro effects of tobacco products on PBMCs, however, Takizawa, H. et al. report that TGF-β1 gene expression was elevated in the alveolar walls of smokers [29]. Cigarette smoke extract has also been found to induce expression of TGF-β in alveolar epithelial cells [30], and such induction was found to be dose dependent in rats [31]. These findings confirm that cigarette smoking acts on many different tissues, indicating that changes caused by smoking probably start at various locations at different times.

While oxidative stress is known to contribute to the induction and persistence of TGF-β1-induced pulmonary fibrosis [32], cigarette smoke has been found to inhibit the ability of human bronchial epithelial cells to participate in repair processes [33]. The PBMC used in our study might only partially reflect the changes occurring in cigarette smoke-targeted organs. As noticed by van Leeuwen, D.M. et al. [34], in human volunteers it is not possible to investigate biological effects in cells from target organs like lung and liver [35]. It is still unknown whether effects at the gene expression level observed in PBMC reflect those in target organs.

In our study, CSI-induced TGF-β and TIMP-1 mRNA expression was decreased by apocynin. TGF-β is known to require ROS, which are inhibited by apocynin [36,37]. TIMPs are in turn tightly controlled by TGF-β [38,39]. Hence, ROS inhibition might influence both genes in a similar way. Apocynin was found to be effective in decreasing CSI-induced TIMP-1 and TGF-β in the PBMCs of smoking subjects (*p* < 0.05). It has been reported that TIMP-1 plays a key role in the regulation of ECM accumulation. Excessive TIMP-1 expression markedly reduces MMP activity, resulting in the accumulation of collagen, deposition of other ECM elements and consequent fibrosis [40].

TIMP-1 and MMP-9 expression has been found to be higher in COPD [41], suggesting that short-term smoking results in changes that may lead to COPD. Tollefson, A.K. et al. suggest that the mechanisms underlying the host defense against cigarette smoke-induced oxidative damage and subsequent development of COPD include endogenous oxidases and antioxidant enzymes [42]. Hence, apocynin, being an NADPH inhibitor, might have the potential to limit these changes.

The evidence consistently shows that smoking decreases circulating plasma levels of antioxidants [43,44,45]. The second part of our study evaluated the differences in superoxide dismutase 1, TBAR, and thiol group levels between short-term smokers and those who had never smoked to assess the oxidative condition of PBMCs and their antioxidant capability.

TBARs were significantly higher in PBMC and serum of smokers, which are consistent with previous studies [46,47,48]. Additionally, apocynin significantly decreased CSI-induced TBARs in smokers (*p* < 0.05). Similarly, apocynin increased the levels of thiol groups in PBMC after CSI treatment. It is widely known that apocynin not only inhibits NADPH but also acts as an antioxidant [49,50,51,52]. Apocynin has been demonstrated to be effective in the reduction of H_2_O_2_ and NO_2_ in COPD patients with no adverse events [53]. However, our current study suggests that apocynin might also contribute to strengthening the antioxidant defense. Liu, J.J. et al. found apocynin to have a clear protective effect against cardiac remodeling in a rat model, which was associated with a significant increase in SOD activity and decrease in MDA concentration. Treatment with apocynin also significantly inhibited deposition of collagen and reduced the level of MMP-2 [54].

The increase in MDA level was found to be significantly attenuated by treatment with apocynin in angiotensin II-induced hypertensive mice [55], and that antioxidant treatment, including apocynin, significantly attenuated gene expression of TGF-β, type-I collagen, and TIMP-1 in the heart of a rat model [56]. Studies indicate that apocynin presents a wide spectrum of properties, which may possibly be incorporated in COPD therapy.

The present study also investigates the expression of genes which contribute to airway remodeling and the oxidative/antioxidative properties of PBMC in both groups (Table 2). A strong correlation was found between SOD1 levels and arginase mRNA expression in smokers (r = 0.91, *p* < 0.05), but not in nonsmokers (r = 0.3, *p* > 0.05). Moreover, in the smoking group, a strong correlation was also observed between SOD1 concentrations and MMP-9 mRNA expression (r = 0.83, vs. −0.3 in never smoking subjects, *p* < 0.05). These results might be interpreted as relation—the higher mRNA expression of the gene (and thus—the lower ΔCt), the lower SOD1 levels. Only the smoking group demonstrated such strong correlations were revealed.

These results confirm that cigarette smoke ingredients activate biological pathways, which are not only involved in remodeling but also concern oxidative defense. Whether this relates to the fact that PBMCs were used, remains unclear. Nevertheless, considering the scope of the devastation caused by smoking, it is possible that the changes may be noticeable in peripheral cells.

It is widely known that cigarette smoke decreases the ability of cells to defend against oxidative stress. However, only a few ingredients of the smoke were used in the present study, which may be regarded as a limitation to the study. In addition, it is not certain that the blood cells of smokers may be exposed to such precise concentrations of ingredients as used in the study. Therefore, our results should be interpreted with caution. We are also aware that with the limited size of the study groups, it cannot be excluded that the changes in the analyzed biomarkers might be dependent on other factors.

In conclusion, the study indicates that short-term smoking evokes changes that may lead to serious disorders, and possibly COPD. These changes are strictly connected with the oxidative status of cells, and more importantly—they, are so serious as to be visible in peripheral blood cells.

## 5. Conclusions

Only a few years of smoking results in changes as deep as alterations of expression in blood cells. Though the precise mechanism connecting cigarette smoking and airway remodeling is not known, the changes appearing in short-term smokers may well influence the oxidative status of the cells. Interestingly, apocynin seems to alleviate these changes, indicating that ROS influence arginase and TGF-β expression.

## Figures and Tables

**Table 1:** Characteristics of study participants.

Variable	Smokers (*n* = 8)	Nonsmokers (*n* = 8)
Women	4	5
Men	4	3
Age (yrs) [min-max]	27 ± 6	25 ± 6
BMI	23 ± 2	22 ± 3
Cigarettes per day	13 ± 8	NA
Years smoking	6 ± 5	NA
Smoking Pack-Years	4 ± 3	NA
FEV1 [%]	95	101
FVC [%]	93	99

**Table 2:** Trypan blue exclusion test results.

Cells Treatment	Cell Viability [%]	
	Smokers	Nonsmokers
Control	95	96
Apocynin	94	94
CSI	86	87

**Table 3:** Pearson correlation coefficient results comparing expression of chosen genes (deltaCt) and oxidative/antioxidative parameters of the cells.

	*TGF-β*		*ARG*		*MMP-9*		*TIMP-1*	
	Nsm	Sm	Nsm	Sm	Nsm	Sm	Nsm	Sm
**TBARs PBMC**	−0.13	−0.2	0.3	0.2	−0.5	0.14	−0.03	0.09
**TBARs serum**	−0.01	−0.47	0.1	0.1	−0.5	−0.21	0.2	0.07
**thiols**	0.18	0.38	0.5	0.3	−0.02	−0.01	0.2	−0.1
**SOD1**	0.6	0.13	0.3	0.91 *	−0.3 *	0.83 *	−0.1	−0.2
**SOD1 mRNA**	0.4	0.02	0.38 *	−0.6 *	0.33	−0.72	0.3	0.1

Nsm—non-smoking group; Sm—smoking group; *—*p* < 0.05. “−“ means negative correlation. * means statistical significance.
